# Optimal Inhibition of Choroidal Neovascularization by scAAV2 with VMD2 Promoter-driven Active Rap1a in the RPE

**DOI:** 10.1038/s41598-019-52163-z

**Published:** 2019-10-31

**Authors:** Haibo Wang, Eric Kunz, Gregory J. Stoddard, William W. Hauswirth, M. Elizabeth Hartnett

**Affiliations:** 1 John A Moran Eye Center, Salt Lake City, UT 84132 USA; 2 Department of Internal Medicine, University of Utah, Salt Lake City, UT 84132 USA; 3 Department of Ophthalmology, College of Medicine, University of Florida, Gainesville, FL 32610 USA

**Keywords:** Translational research, Experimental models of disease

## Abstract

Age-related macular degeneration (AMD) is a multifactorial chronic disease that requires long term treatment. Gene therapy is being considered as a promising tool to treat AMD. We found that increased activation of Rap1a in the retinal pigment epithelium (RPE) reduces oxidative signaling to maintain barrier integrity of the RPE and resist neural sensory retinal angiogenesis from choroidal endothelial cell invasion. To optimally deliver constitutively active Rap1a (CARap1a) into the RPE of wild type mice, self-complementary AAV2 (scAAV2) vectors driven by two different promoters, RPE65 or VMD2, were generated and tested for optimal active Rap1a expression and inhibition of choroidal neovascularization (CNV) induced by laser injury. scAAV2-VMD2, but not scAAV2-RPE65, specifically and efficiently transduced the RPE to increase active Rap1a protein in the RPE. Mice with increased Rap1a from the scAAV2-VMD2-CARap1a had a significant reduction in CNV compared to controls. Increased active Rap1a in the RPE *in vivo* or *in vitro* inhibited inflammatory and angiogenic signaling determined by decreased activation of NF-κB and expression of VEGF without causing increased cell death or autophagy measured by increased LCA3/B. Our study provides a potential future strategy to deliver active Rap1a to the RPE in order to protect against both atrophic and neovascular AMD.

## Introduction

Age-related macular degeneration (AMD) remains a leading cause of legal blindness in the elderly worldwide^1,2^. Evidence suggests that dysfunction of the retinal pigment epithelium (RPE) precedes both neovascular and atrophic forms of AMD and may be important in the pathogenesis of these end-stage forms of AMD^3^. The RPE is a monolayer of polarized cells that is critically important in retinal homeostasis. The RPE maintains the outer blood-retinal barrier while it regulates nutrient and oxygen delivery to the outer retina and removal of metabolic waste from the photoreceptors. The RPE also produces growth factors at a physiologic level that support the retina and choriocapillaris^3–5^. With aging and increasing pathologic stresses, the RPE can lose the efficiency of these functions. As a result, accumulation of debris within Bruch’s membrane appear as drusen beneath the RPE. As dysfunction progresses, the barrier integrity of the RPE is compromised and stressed RPE releases growth factors at a pathologic level that lead to advanced AMD^6^. Therefore, strategies to maintain or restore functions of the RPE might be potential targets for AMD therapy.

Activation of Rap1a, a GTPase protein, was found to protect the barrier function of the RPE from inflammatory stress^7^. In a murine model of laser-induced choroidal neovascularization (CNV), Rap1 activity was decreased in the RPE and choroid tissues, but delivery of intravitreal 8-CPT-2Me-cAMP to activate endogenous Rap1 inhibited CNV induced by laser^8,9^. We looked into gene therapy as an approach to target the RPE specifically and potentially reduce the number of treatments required by intravitreal delivery. Because of lack of pathogenicity, the low immunogenicity, relatively long-term transgene expression compared to intravitreal neutralizing antibodies or pharmacologic agents, and high transduction efficiency, vectors of adenovirus-associated virus (AAV) are becoming promising tools to treat retinal degeneration^10–12^. To increase active Rap1a in the RPE, a constitutively active Rap1a (CARap1a) was delivered in a self-complementary AAV2 (scAAV2) viral vector driven by the RPE65 promoter (scAAV2-RPE65) and was found to reduce experimental CNV in Rap1b deficient mice but not in wild type mice^7^.

The insufficient reduction of CNV in wild type mice by the scAAV2-RPE65 delivered CARap1a was predicted to be related to the weak transcriptional activity of the RPE65 promoter. To test this possibility, the RPE65 promoter was compared to another specific promoter of the RPE, vitelliform macular dystrophy-2 (VMD2)^13,14^. We compared the two promoters, RPE65 and VMD2, in driving the expression of Rap1a in the RPE and in reducing experimental CNV in wild type mice. In this study, the effect of the expression of exogenous CARap1a was also evaluated and compared in eyes treated with the two different promoters, RPE65 and VMD2.

## Results

### Generation of self-complementary adeno-associated virus 2 (scAAV2) driven by VMD2 promoter

**Figure 1:**
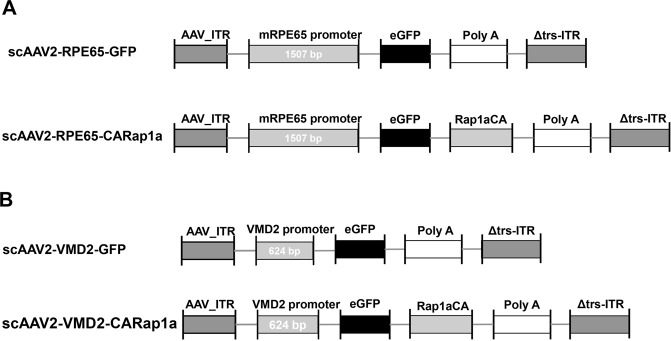
Diagrams of self-complementary adeno-associated virus 2 (scAAV2) to deliver constitutively active Rap1a (CARap1a) or only GFP driven by (**A**) an RPE65 promoter (scAAV2-RPE65-GFP-CARap1a and scAAV2-RPE65-GFP) or (**B**) a VMD2 promoter (scAAV2-VMD2-GFP-CARap1a and scAAV2-VMD2-GFP).

A self-complementary adeno-associated virus 2 (scAAV2) with a green fluorescent protein (GFP) tag was used in this study. The scAAV2 driven by a murine RPE65 promoter expressing either GFP alone or GFP and active Rap1a (CARap1a) (Fig. 1A) was generated as described previously^7^. To compare the transcriptional activity of RPE65 and VMD2 promoters in delivering active Rap1a in RPE, a murine VMD2 promoter was cloned into scAAV2 vector to replace the RPE65 promoter, driving either GFP or GFP and active Rap1a (CARap1a) (Fig. 1B).

### *In vivo* analysis of scAAV2 transduction and Rap1 expression

**Figure 2:**
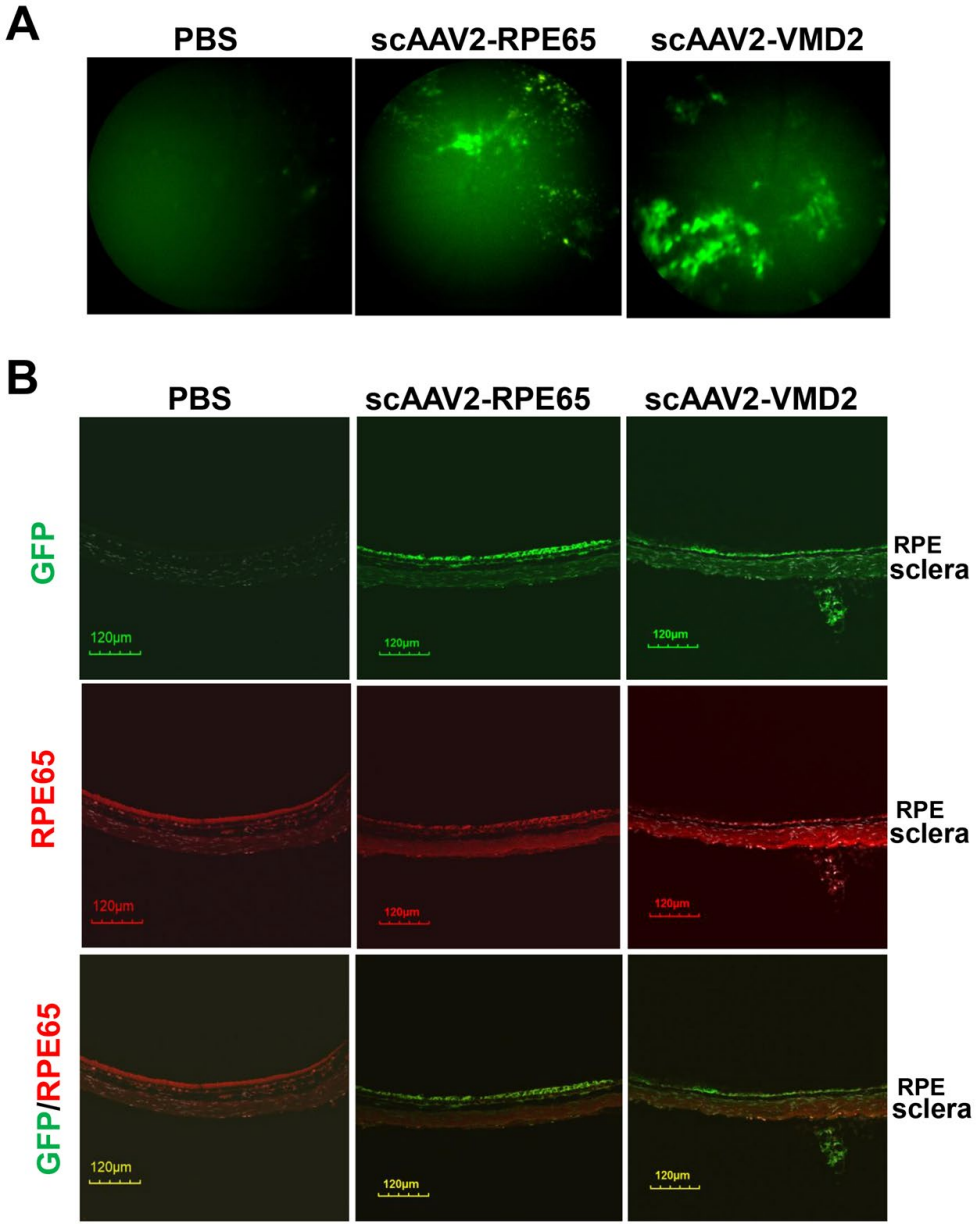
*In vivo* analysis of scAAV2 transduction in RPE of wild type mice. (**A**) Micron IV retinal imaging of GFP and (**B**) immunostaining of GFP and RPE65 in retinal cryosections of wild type mice treated with PBS or 5 weeks after injection of scAAV2-RPE65-GFP or scAAV2-VMD2-GFP vectors at dose of 5 × 10^8^ viral particle/µl.

**Figure 3:**
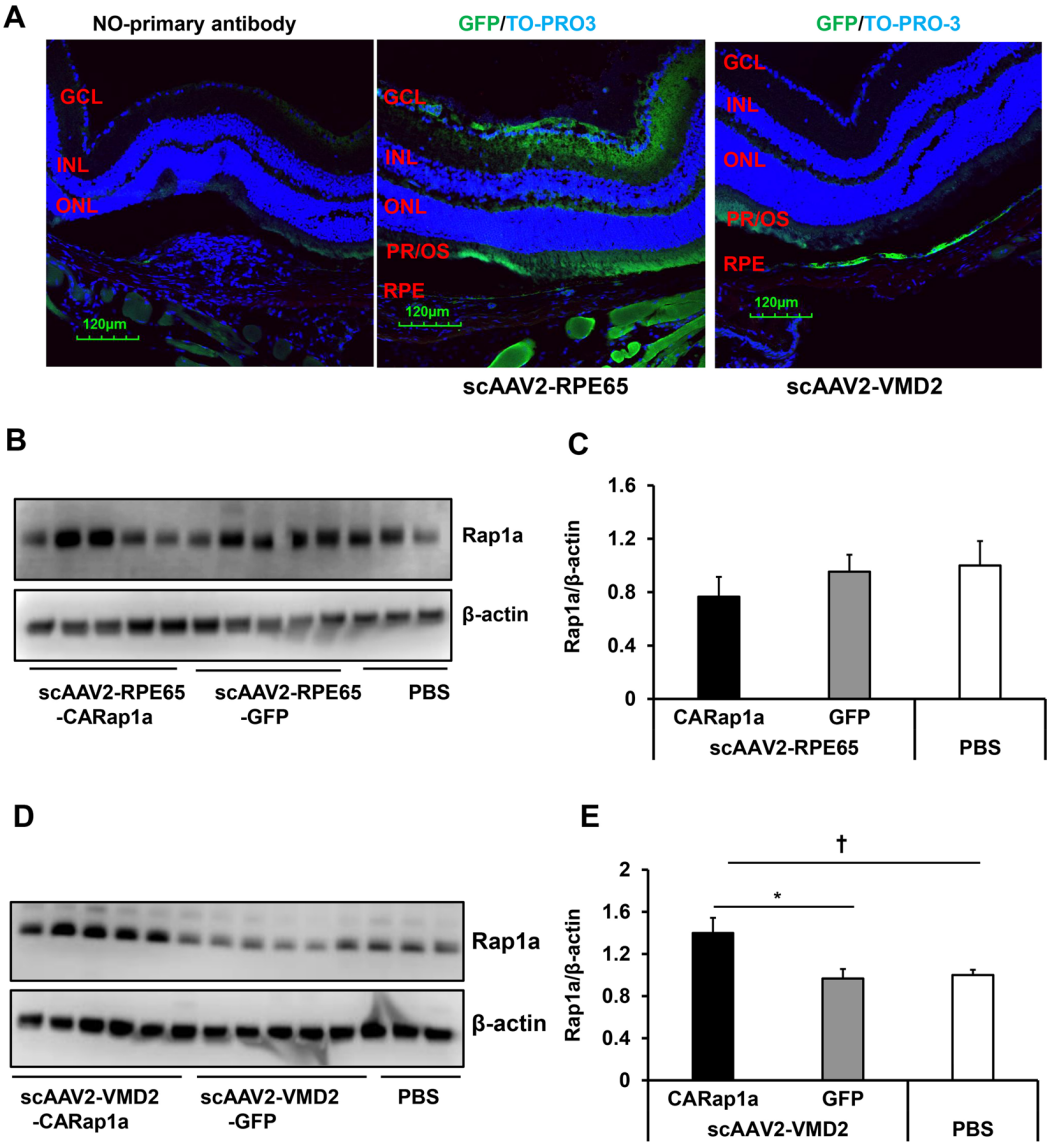
scAAV2-VMD2 vector shows more specific GFP transduction and greater Rap1 expression in the RPE. (**A**) IHC of GFP in retinal cryosections (Blue: TO-PRO-3; Green: GFP) (**B–E**) western blots of Rap1a and β-actin in RPE/choroids (**B–D**), (representative gel images and C and E, quantification of densitometry) of wild type mice injected with either (**B,C**) scAAV2-RPE65 or (**D**,**E**) scAAV2-VMD2 or PBS (*p < 0.05 *vs*. scAAV2-VMD2-GFP; ^†^p < 0.05 vs. PBS n = 5–6.

To determine the viral transduction efficiency, scAAV2-RPE65 or scAAV2-VMD2 virus at 5 × 10^8^ viral particle/µl was delivered into the subretinal space of both eyes of 6-week-old wild type mice. Viral transduction was determined by GFP visualization using a Micron IV retinal live imaging system at week 5 after injection. As shown in Fig. 2A, both scAAV2-RPE65 and scAAV2-VMD2 showed GFP expression, whereas PBS-injected eyes did not show GFP expression. To confirm the viral transduction targeted the RPE, GFP positive eyes were harvested and the RPE/choroid cryosections were immunolabeled with GFP and RPE65 antibodies. Both scAAV2-RPE65 and scAAV2-VMD2 virus treated eyes showed GFP colabeling with RPE65 (Fig. 2B), suggesting both viral vectors can transduce the RPE of wild type mice. To further determine the specificity of AAV2 viral transduction, GFP immunostaining was performed in whole retinal cryosections. In scAAV2-RPE65 treated retina, GFP immunolabeling was not only located in the layer of the RPE, but also in the retinal ganglion cells and photoreceptor outer segments (PR/OS); however, in scAAV2-VMD2 treated retina, GFP immunolabeling was mainly found in the layer of the RPE and the PR/OS (Fig. 3A) showing that the scAAV2-VMD2 has a more specific pattern of transgene expression related to the more restricted RPE cell specific activity of the VMD2 promoter. By western blots using an antibody to total Rap1, we next determined Rap1a protein levels in RPE/choroid tissues from GFP-positive eyes 5 weeks after subretinal injections. As shown in Fig. 3D,E Rap1a protein was significantly increased in scAAV2-CARap1a treated RPE/choroid lysates compared to scAAV2-VMD2-GFP. However, eyes treated with scAAV2-RPE65-CARap1a did not show increased Rap1a protein compared to scAAV2-RPE65-GFP (Fig. 3B,C). The data in Figs 2 and 3 provide evidence that both scAAV2-RPE65 and scAAV2-VMD2 transduced the RPE of wild type mice, but only scAAV2-VMD2 efficiently drove Rap1a expression.

### Expression of active Rap1a in RPE delivered by scAAV2-VMD2 reduces CNV in wild type mice

**Figure 4:**
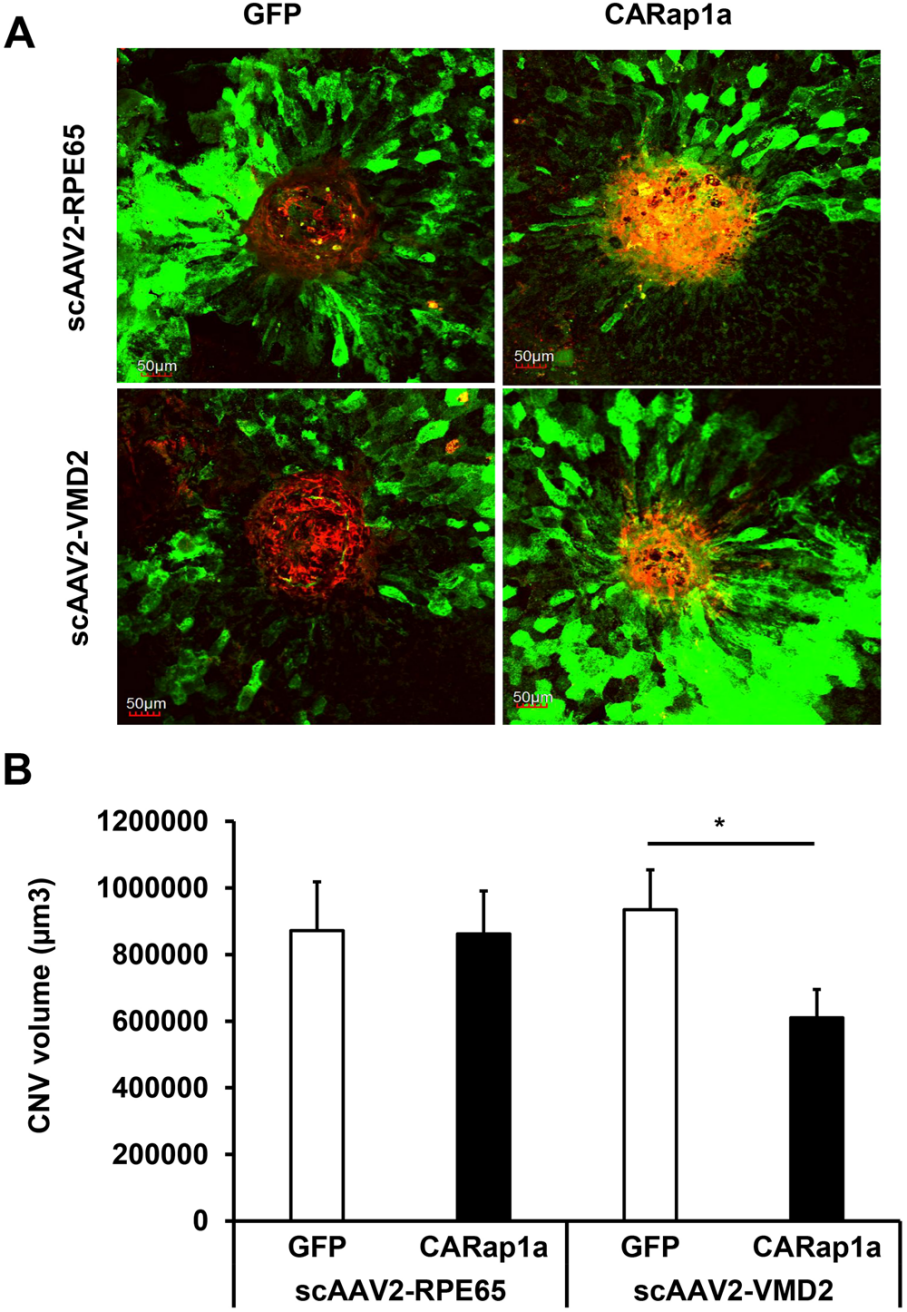
Expression of active Rap1a in RPE by scAAV2-VMD2-CARap1a reduces choroidal neovascularization (CNV) in wild type mice in a laser induced CNV model. (**A**) Representative images of RPE/choroid flat mounts and (**B**) quantification of CNV lesion (*p < 0.05 vs. scAAV2-VMD2, n = 40 spots from 12 mice).

We previously reported that expression of active Rap1a in RPE by scAAV2-RPE65 reduced laser-induced CNV in Rap1 deficient mice but not in wild type mice^7^. We, therefore, determined if increased Rap1a in RPE by scAAV2-VMD2 would reduce CNV induced by laser in wild type mice comparing outcomes to mice with subretinal injections of scAAV2-RPE65 (Supplementary Figure 2A). To compare each experimental vector and its respective control, we used ANOVA analysis, which considered each CNV lesion as an individual data point. Consistent with our previous findings, scAAV2-RPE65-CARap1a did not reduce CNV compared to scAAV2-RPE65-GFP^7^; however, compared to scAAV2-VMD2-GFP, scAAV2-VMD2-CARap1a significantly reduced CNV by ANOVA analysis (p = 0.026) (Fig. 4). To further confirm if each CNV lesion could be considered as an individual data point, we additionally ran the statistical analysis using a mixed effects linear regression to compare scAAV2-VMD2-CARap1a and scAAV2-VMD2-GFP. The amount of correlation among the spots within the same eye was measured with the intraclass correction coefficient (ICC), which was ICC = 0.16, 95% CI (0.02, 0.53), suggesting that an ordinary two sample ANOVA would not be appropriate, as it requires that all observations, or spots, be independent. By a mixed effects linear regression analysis, the mean ± SE in the scAAV2-VMD2-CARap1a group was 619,928 ± 124,932, and in the control scAAV2-VMD2-GFP was 952,091 ± 124.932; compared to scAAV2-VMD2-GFP, there was a statistically significant difference (mean difference, 336,162, 95% CI: 2,454 to 647,778; p = 0.05). The mixed effects linear regression  analysis providied  support  that scAAV2-VMD2-CARap1a significantly reduced CNV in wild type mice.

### Expression of active Rap1a in RPE reduces inflammatory signaling and VEGF expression in RPE/choroid tissues

**Figure 5:**
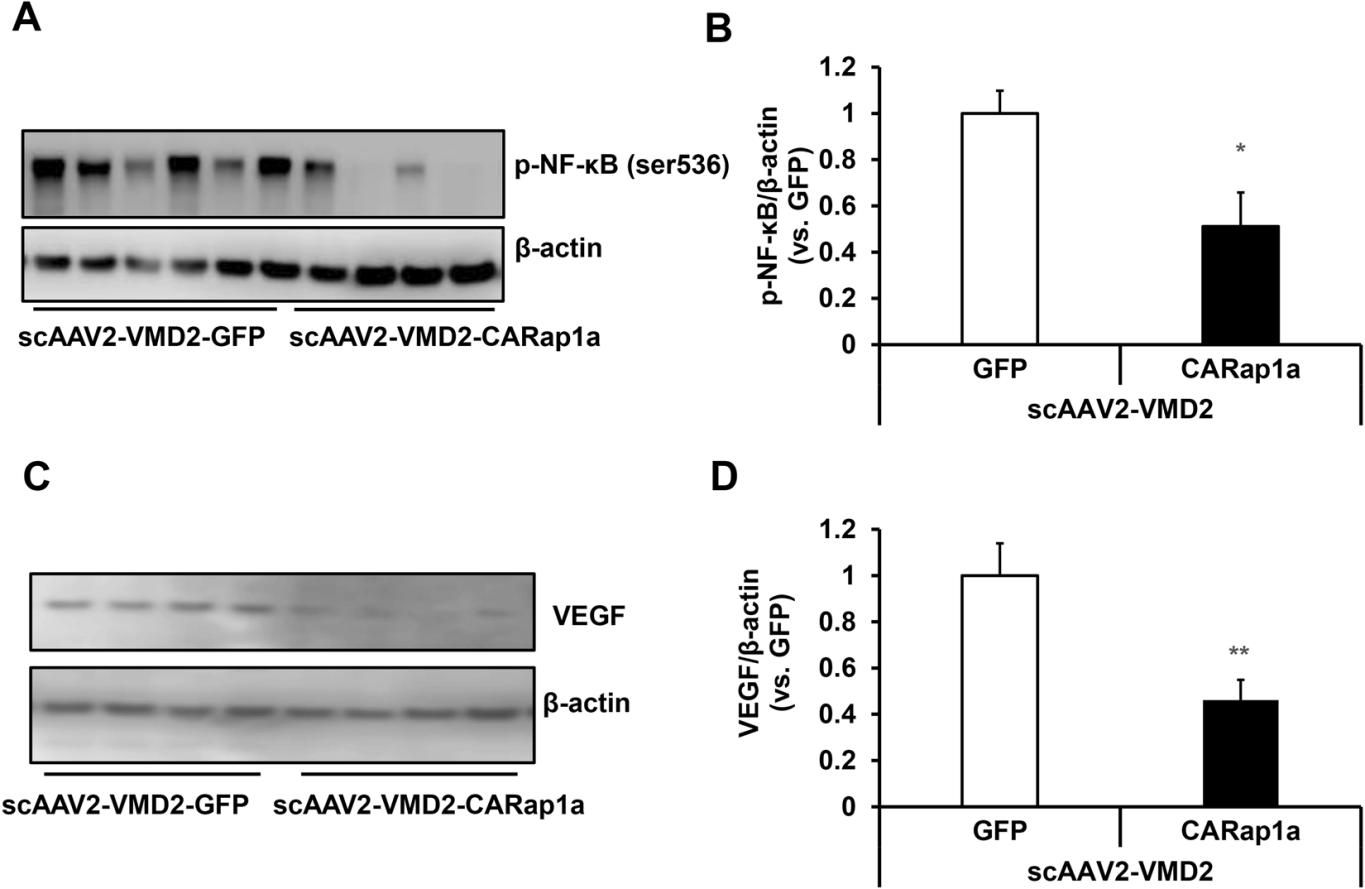
Expression of active Rap1a in RPE by scAAV2-VMD2-CARap1a reduces inflammation and VEGF in RPE/choroids. Western blots of (**A,B**) phosphorylated NF-κB (p-NF-κB) and (**C,D**) VEGF in RPE/choroids of scAAV2-VMD2 injected wild type mice 7 days after laser treatment (**A–C**), representative gel images and (**B–D)**, quantification of densitometry; *p < 0.05, **p < 0.01 vs. scAAV2-VMD2-GFP; n = 6–8.

Both inflammation and VEGF signaling are involved in the pathogenesis of AMD^2^. We previously found that laser treatment significantly increased TNFα in RPE/choroid tissues, and inhibition of TNFα by an intravitreal neutralizing antibody reduced CNV^15^. We, therefore, determined if increased active Rap1a in the RPE by scAAV2-VMD2-CARap1a would reduce inflammation by employing TNFα mediated signaling as a test example. Phosphorylation of nuclear factor kappa of activated B cells (NF-κB), a downstream effector of TNFα, was measured in RPE/choroid lysates by western blots. As shown in Fig. 5A,B, phosphorylated NF-κB (p-NF-κB) was significantly decreased by scAAV2-VMD2-CARap1a compared to scAAV2-VMD2-GFP. In the same tissue lysates, VEGF protein was also significantly decreased by scAAV2-VMD2-CARap1a compared to scAAV2-VMD2-GFP (Fig. 5C,D).

### Expression of active Rap1a in RPE reduces caspase 3 and LC3A/B in RPE/choroid tissues

**Figure 6:**
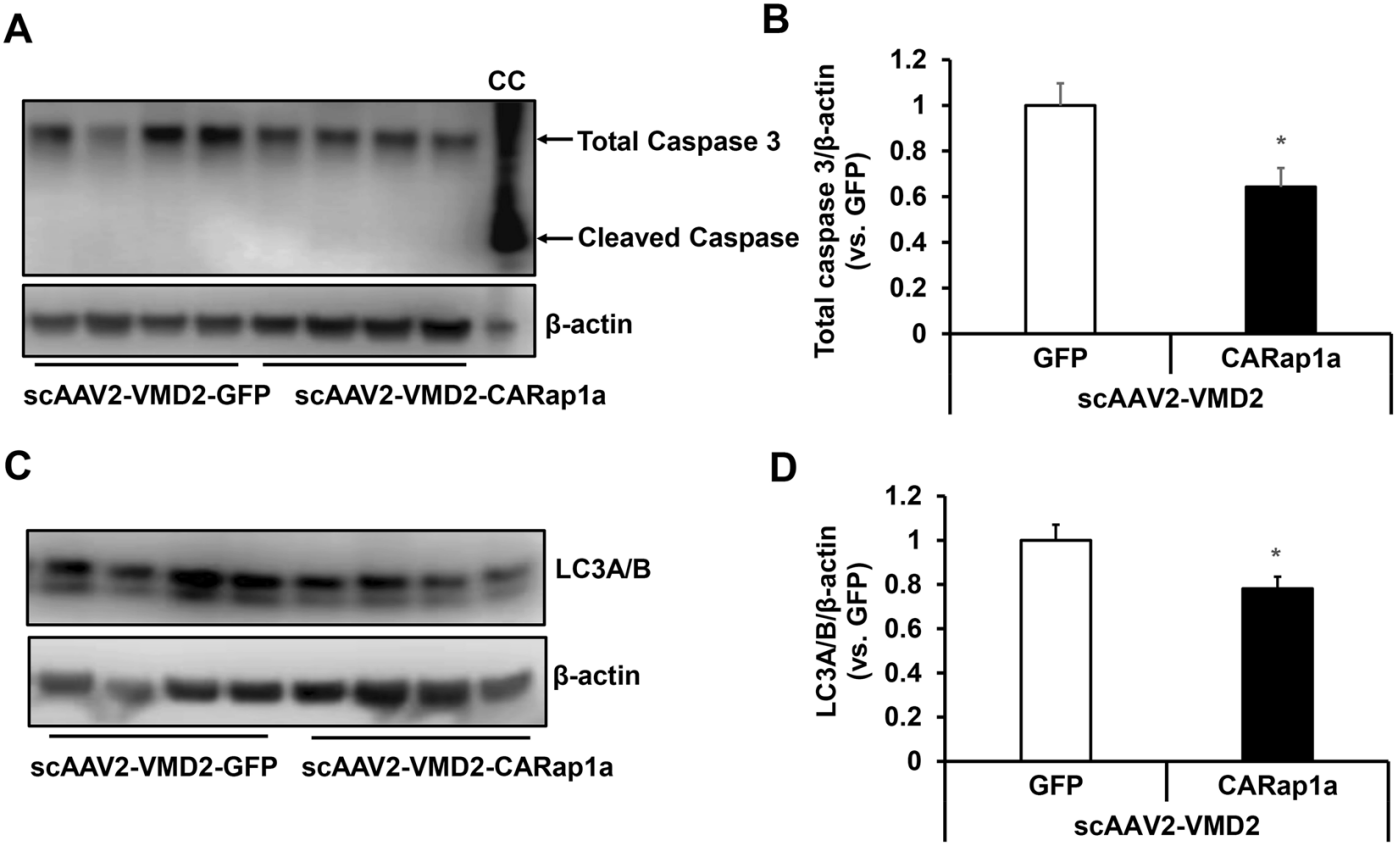
Expression of active Rap1a in RPE by scAAV2-VMD2-CARap1a does not activate apoptosis and autophagy. Western blots of (**A,B**) caspase 3 and (**C,D**) LC3A/B in RPE/choroids of scAAV2-VMD2 injected wild type mice 7 days after laser treatment (**A–C**), representative gel images and (**B**–**D**), quantification of densitometry; *p < 0.05 vs. scAAV2-VMD2-GFP; n = 5–6; CC, cytochrome C treated cell lysate.

The results in Figs 3 and 4 provide evidence to support the hypothesis that the scAAV2-VMD2 vector efficiently drove active Rap1a expression specifically in the RPE and reduced CNV in wild type mice. We next determined if increased active Rap1a in RPE by scAAV2-VMD2-CARap1a would overwhelm RPE homeostasis, since delivering a protein may overwhelm the cell’s ability to maintain its viability. Caspase 3 and cleaved caspase 3, an apoptotic maker, and LC3A/B, an autophagic regulator, were measured in RPE/choroid lysates from scAAV2-VMD2 treated eyes. As shown in Fig. 6, compared to scAAV2-VMD2-GFP, total caspase 3 (Fig. 6A,B) and LC3A/B (Fig. 6C,D) in RPE/choroid tissues were significantly decreased by scAAV2-VMD2-CARap1a. Cleaved caspase 3 was not detected in RPE/choroid tissues from either group. Taken together, the data shown in Fig. 6 suggest that expression of active Rap1a in RPE by scAAV2-VMD2 does not cause activation of caspase 3 and excessive activation of autophagy.

### Expression of active Rap1a in RPE reduces VEGF, TNFα-induced NF-κB activation and LC3A/B without causing cell death

**Figure 7:**
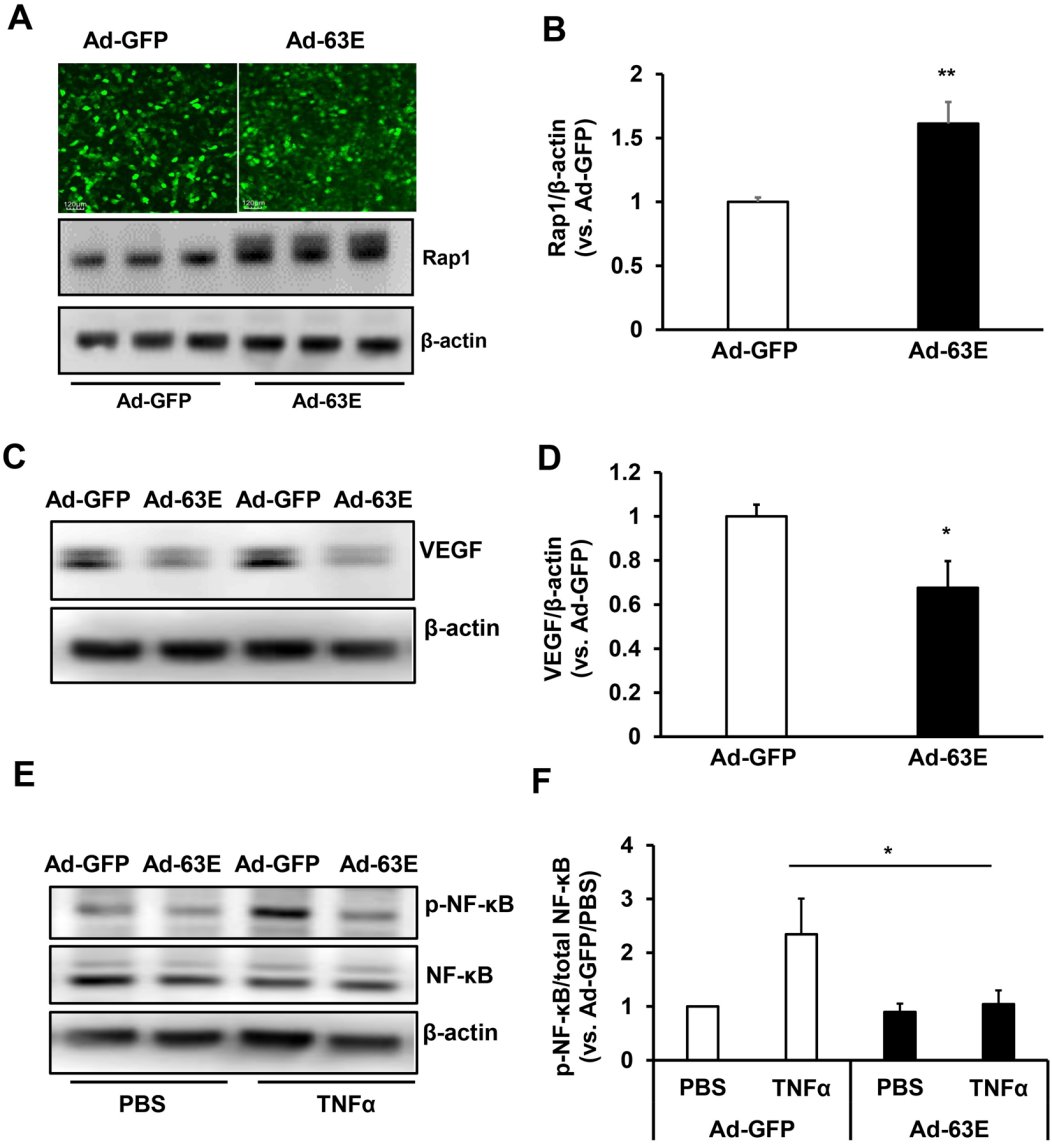
Expression of active Rap1a in RPE by adenovirus transduction reduces VEGF and NF-κB activation. Western blots of (**A,B**) Rap1, (**C,D**) VEGF protein and (**E,F**) phosphorylated NF-κB (p-NF-κB) and total NF-κB in human RPE transduced with adenovirus expressing GFP (Ad-GFP) or GFP and constitutively active Rap1a (Ad-63E) (**A,C,E**), representative gel images and (**B,D,F**), quantification of densitometry *p < 0.05, **p < 0.01 vs. Ad-GFP; n = 3.

**Figure 8:**
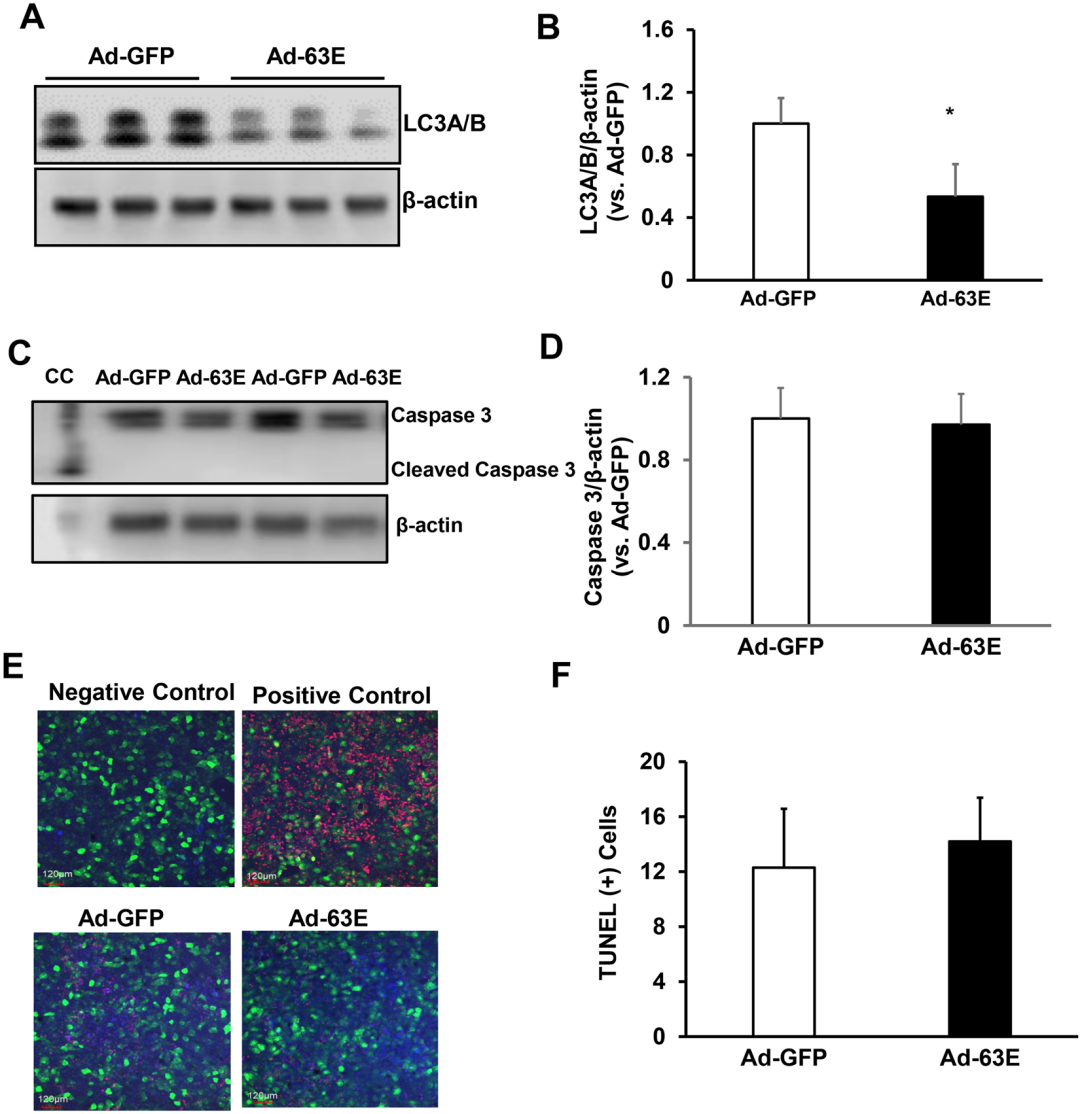
Expression of active Rap1a in RPE by adenovirus transduction does not increase autophagy and cell death. Western blots of (**A,B**) LC3A/B protein and (**C,D**) caspase 3 and cleaved caspase 3; and (J-K) TUNEL staining in human RPE transduced with adenovirus expressing GFP (Ad-GFP) or GFP and constitutively active Rap1a (Ad-63E) (**A and C**), (representative gel images and (**B and D)**, quantification of densitometry *p < 0.05 vs. Ad-GFP; n = 3; CC in C refers to cytochrome C treated cell lysate.

To further assess the effects of active Rap1a on VEGF expression, activation of inflammatory signaling, autophagy and cell death, we performed a series of experiments in cultured human RPE transduced with adenovirus expressing the Rap1a Q63E mutant that constitutively activates Rap1a (Ad-63E) or control adenovirus expressing only GFP (Ad-GFP). The viral transduction was monitored by GFP visualization. Forty-eight hours after viral transduction, about 80% of the RPE were GFP positive (Fig. 7A), and total Rap1 protein measured by western blots was increased in RPE transduced with Ad-63E compared to Ad-GFP (Fig. 7A,B). In Ad-63E transduced RPE, VEGF protein (Fig. 7C,D), TNFα-induced p-NF-κB (Fig. 7E,F) and LC3A/B (Fig. 8A,B) were significantly reduced compared to Ad-GFP. Cleaved caspase 3 was not detected in either Ad-GFP or Ad-63E transduced RPE cells and total caspase 3 was not significantly reduced by Ad-63E (Fig. 8C,D). To further determine if expression of exogenous active Rap1a would induce cell death, TUNEL staining was performed in RPE 48 hours after viral transduction. As shown in Fig. 8E,F, Ad-63E transduction did not increase TUNEL positive cells compared to Ad-GFP. Taken together, the data shown in Figs 7 and 8 provide further support that the expression of exogenous active Rap1a reduces inflammation and VEGF without increasing cell death and autophagy.

## Discussion

AMD is a complex and multifactorial disease characterized by irreversible central vision impairment. Although the pathophysiologic steps of AMD are still being elucidated, extensive evidence supports the concept that the progression of AMD is affected by interactions of aging, genetic and environmental factors^2,16–19^. These interactions trigger signaling pathways involving inflammation, oxidative stress, cell death mechanisms and angiogenesis in the RPE and choroidal endothelial cells and lead to vision loss from cell degeneration and CNV^2,17^. Treatments targeting vascular endothelial growth factor (VEGF) have greatly improved clinical outcomes in neovascular AMD; however, vision improvement only occurs in less than half of patients treated for neovascular AMD^20,21^, and treatments remain inadequate for atrophic AMD.

Gene therapy has been gaining much attention in treating AMD as it provides the potential for long-term treatment, which would reduce the number of repeated treatments associated with local delivery with intravitreal injections of anti-VEGF agents. Gene therapy also offers possibilities to target particular cells by using cell specific promoters. Using a gene therapy approach, we previously reported that the expression of exogenous active Rap1a in the RPE by a scAAV2-RPE65 vector significantly reduced laser-induced CNV in Rap1b deficient mice but not in wild type mice^7^. In this study, we tested another specific promoter of the RPE, VMD2, in driving expression of active Rap1a in RPE in wild type mice, and the effects were compared with the scAAV2-RPE65. Our study showed that the scAAV2-VMD2 vector efficiently drove active Rap1a expression in the RPE of wild type mice and transduction of scAAV2-VMD2 had higher specificity in the RPE compared to sc-AAV2-RPE65. The scAAV2-RPE65 promoter did not increase active Rap1a in RPE in wild type mice. We also found that scAAV2-VMD2-CARap1a treated mice with increased active Rap1a in the RPE had a significant reduction in CNV compared to those treated with the control vector scAAV2-VMD2-GFP. The findings from this study support the hypothesis that VMD2 has a stronger activity in driving active Rap1a expression in the RPE and increased active Rap1a is able to reduce CNV in wild type mice. Other studies have also reported RPE specific transduction of other transgenes with the VMD2 promoter-driven AAV vector and showed reduction of laser-induced CNV in mice^22,23^.

The crosstalk and feed-back loops involving inflammation, oxidative signaling and angiogenesis are implicated in the pathogenesis of AMD^2^. We previously tested the role of inflammation in experimental CNV using the inflammatory cytokine, TNFα, as an example of a cytokine that has been associated with AMD and CNV^24,25^. We reported that intravitreal TNFα contributed to experimental CNV through a mechanisms involving reactive oxygen-triggered VEGF production in the RPE^15,26^. Furthermore, activation of Rap1a in the RPE reduced the generation of reactive oxygen species^9,26^. Here, our study showed that scAAV2-VMD2-CARap1a treated eyes had a significant reduction in VEGF and NF-κB phosphorylation in RPE/choroid tissues compared to control scAAV2-VMD2-GFP. These findings were further supported using cultured human RPE, in which increased active Rap1a by adenoviral transduction significantly reduced VEGF and p-NF-κB. However, we did not observe similar effects in scAAV2-RPE65-CARap1a treated eyes. These results support the hypothesis that increased active Rap1a in RPE reduced CNV by reducing VEGF. The reduction in inflammatory signaling can reduce stimuli contributing to CNV, as we found previously using TNFα as an inflammatory cytokine^15,26^, but may also reduce atrophic AMD by interfering with processes leading to cell death^27–29^.

One concern with introducing a protein to be protective is the risk of overwhelming the cell’s natural abilities to manage proteins^30^. Autophagy is one of the mechanisms by which cells deal with stresses to maintain cellular homeostasis. Through autophagy, misfolded or aggregated proteins and damaged cellular organelles that form in response to overwhelmed cellular stresses can be degraded^31^. Therefore, increased autophagy can indirectly reflect increased cellular stresses. To determine if introduction of active Rap1a through gene therapy would cause cellular stress to trigger cell death by apoptosis or excessive activation of autophagy, we evaluated apoptosis by measuring cleaved caspase-3 and autophagy by LC3A/B following induced expression of exogenous active Rap1a in RPE by scAAV2-VMD2. In the laser-induced CNV model, compared to scAAV2-VMD2-GFP, scAAV2-VMD2-CARap1a did not increase cleaved caspase-3 but reduced caspase 3 and LC3A/B in RPE/choroid tissues in wild type mice. We also observed that increased Rap1a activity in human RPE cells *in vitro* reduced LC3A/B without increasing caspase 3 activation and cell death determined by TUNEL staining.

In conclusion, the VMD2 promoter targeted RPE more specifically and increased Rap1a expression compared to the RPE65 promoter. Increased active Rap1a in the RPE by VMD2 promoter reduced three effectors associated with advanced AMD: VEGF, activated NF-κB and LC3A/B. Activation of Rap1a may protect against AMD-related stimuli leading to inflammation and angiogenesis and maintain RPE integrity and function. scAAV2-VMD2 vector may be an efficient and safe tool to deliver genetic materials to the RPE but long-term effects in other models of AMD, including those modeling geographic atrophy, will require additional study.

## Materials and Methods

### Animals

Six-week-old C57BL/6J male and female mice were purchased from the Jackson Laboratory (Bar Harbor, ME). As described previously^7^, all animal procedures were performed according to Guide for the Care and Use of Laboratory Animals of the University of Utah) and the Association for Research in Vision and Ophthalmology Statement for the Use of Animals in Ophthalmic and Vision Research, and approved by IACUC and the Institutional Biosafety Committee of the University of Utah. Ketamine (100 mg/kg) and xylazine (20 mg/kg) were used for animal anesthesia, and cervical dislocation was performed for animal euthanasia under anesthesia.

### Construction of RPE65 or VMD2 promoter driven Self-complementary Adeno-associated Virus 2

The self-complementary adeno-associated virus 2 (scAAV2) vector driven by the murine RPE65 promoter (1507 bp) (scAAV2-RPE65) was generated by the University of North Carolina Vector Core (Chapel Hill, NC) as described previously^7^. The scAAV2-REP65 construct contains synthetic sequences of the constitutively active human Rap1a Q63E mutant (CARap1a) (scAAV2-RPE65 -CARap1a) was used as an experimental vector and the sc-AAV2-RPE65 construct without CARap1a sequences was used as a control vector (scAAV2-RPE65-GFP). To compare the transduction efficiency and specificity of the RPE65 and VMD2 promoters, scAAV2 vectors driven by the murine VMD2 promoter (624 bp) were generated by the Powell Gene Therapy Center (University of Florida, Gainesville, FL). The sequences of CARap1a were cloned into the scAAV2-VMD2 as scAAV2-VMD2-CARap1a-GFP, and the scAAV2-VMD2-GFP vector was used as a control.

### Subretinal Injections, Micron IV live Imaging and Laser-induced CNV model

Six-week-old mice received bilateral injections of 1 µl of scAAV2 (diluted in fluorescein and PBS to 5 × 10^8^ viral particles) as described previously^7^. Live imaging using the Micron IV retinal imaging system (Phoenix Research Laboratories, Inc., Pleasanton, CA) was used to monitor viral transduction by visualizing GFP fluorescent. Eyes injected with phosphate buffered saline were used as negative controls for GFP visualization by Micron IV.

As described previously^7^, 5 weeks post viral injection, mice received laser to induce CNV. Both eyes of each mouse were first dilated with 1% tropicamide ophthalmic solution. After dilation, mice were treated with 4 spots of laser photocoagulation at 532 nm under anesthesia and each spot was located at about 2 disc diameters from the optic nerve. The laser treatment was performed using the Phoenix Image-Guided Laser System 94 (Phoenix Micron IV, Pleasanton, CA) at settings of ~460 mW intensity and 100 ms duration. Production of cavitation bubbles was considered successful treatment, indicating disrupting the Bruch’s membrane^7^. Seven days post laser treatment, mice were euthanized, and eyes were collected for the analysis of CNV volume and protein.

### Preparation of retinal pigment epithelium (RPE)/Choroid flat mounts and Quantification of CNV volume

As described previously^7^, eyes were fixed in 4% paraformaldehyde (Electron Microscopy Sciences, Hatfield, PA) for 1 hours. The cornea, lens, the vitreous and the retina were then removed, and the posterior eyecups containing the RPE/choroid/sclera were fixed in 4% paraformaldehyde for additional 1 hour. After fixation, the posterior eyecups were blocked in PBS containing 1% bovine serum albumin (BSA) and 0.5% TritonX-100 for 30 mins at room temperature. To label invading choroidal vessels, the eyecups were then incubated with AlexaFluor 568-conjugated Isolectin B4 (1:200, Invitrogen, Carlsbad, CA) overnight at 4 °C. An anti-GFP antibody (1:500, ABCAM, Cambridge, MA) was co-incubated with isolectin B4 to label GFP in RPE. After staining, the eyecup was flattened by cutting radial incisions and flatmounted onto a microscope slide with vectashield mounting medium (Vector Laboratories, Burlingame, CA) for confocal imaging. Confocal z-stack images of each lesion in two channels (568 nm, 488 nm) were acquired using a 20X objective (Olympus Fluoview, Center Valley, PA). Images were imported into IMARIS and lesion volume was measured using the “surfaces” module (Version 9.1.2, Bitplane, Santa Barbara, California, USA). An additional surface was created for the GFP channel (488 nm) to give a rough quantification of VMD2 activation in the immediate area of the lesion. Lesions with obvious hemorrhage or bridging CNV were not included for analysis.

### Immunostaining in retinal cryosections

As described previously^7^, the cornea and lens were removed following 1 hour fixation in 4% paraformaldehyde (Electron Microscopy Sciences, Hatfield, PA). The posterior eyecups were dehydrated in 10% sucrose for two hours followed by 30% sucrose overnight at 4 °C, and were then embedded in optimal cutting temperature (OCT) (Tissue Tek, Hatfield, PA). The frozen tissue blocks were sectioned into 12 μm tissue sections using a cryotome cryostat. After block in 5% normal goat serum in PBS/0.1% TritonX-100 for 1 hour, cryosections (12 μm) were incubated with rabbit anti-GFP (1:200) and RPE65 (1:100) from Abcam (Cambridge, United Kingdom) overnight at 4 °C followed by 1 hour incubation with FITC conjugated goat anti-rabbit secondary antibody (1:200) for GFP and AlexaFluor 594-conjugated goat anti-mouse secondary antibody for RPE65 (Invitrogen, Carlsbad, CA). TO-PRO-3 (1:500, Thermo Fisher Scientific, Waltham, MA) was used to stain nuclei. The sections were mounted in Fluoromount-G (SouthernBiotech, Birmingham AL) after wash in PBS. Images were captured with using a confocal microscope (FV1000, Olympus, Japan).

### Adenoviral transduction in human primary RPE

Human primary RPE (hRPE; Lonza, Walkersville, MD) was grown in retinal pigment epithelial cell basal media (RtEBM, Lonza) and cells at passages 4–6 were used in the experiments. To increase active Rap1a in RPE, the cells were transduced with adenoviral constructs expressing GFP-tagged active Rap1a (Ad-63E) or GFP only (Ad-GFP)^7^. Forty-eight hours post transduction, cells were incubated with recombinant TNF-α (10 ng/ml, R&D Systems, Minneapolis, MN) or PBS for 24 hours.

### TUNEL staining in human RPE

TUNEL staining was performed following manufacturer’s instructions (*In Situ* Cell Death Kit, TMR red; Roche Diagnostics, Indianapolis, IN) as described previously^32^. Human RPE was plated on cell culture coverslips (Thermoscientific, Rochester, NY). After treatment, the cells were first fixed in 4% paraformaldehyde for 1 hour at room temperature. After three washes in PBS, cells were incubated with freshly prepared permeabilization solution (0.1% Triton X-100 in 0.1% sodium citrate) for 2 mins on ice. After permeabilization, some cells were incubated with DNase I (3000 U/ml in 50 mM Tris-HCl, pH 7.5, 1 mg/ml BSA) for 10 minutes at 15–25 °C as positive controls. Cells incubated only with Label Solution without Enzyme Solution were used as negative controls. To identify TUNEL+ cells, cells were incubated with TUNEL reaction mixture (Label Solution and Enzyme Solution Mix in 10:1) for 60 mins at 37 °C in a humidified incubator in the dark. After two washes in PBS, cover slips were mounted with DAPI Fluoromount G. Images were taken under confocal microscope with five random images per coverslip. TUNEL+ cells determined by colabeling with DAPI stained nuclei were quantified, and the mean of TUNEL+ cells in the five images from the same coverslip was used for comparison. There were 5-6 coverslips per condition.

### Protein analysis by western blots

Protein lysates were extracted from RPE/choroid tissues as described previously^7^. Briefly, RPE/choroid tissues were homogenized in radio immunoprecipitation assay buffer (RIPA) (20 mM Tris pH 7.4, 120 mM NaCl, 0.5% sodium deoxycholic acid, 1% Triton X-100, 0.1% SDS, 10% glycerol) with protease inhibitor cocktail (Roche Diagnostics, Indianapolis, IN) and phosphatase inhibitor (2 mM, Sigma-Aldrich, St. Louis, MO) on ice. Protein lysates were collected by centrifuging at 13,000 rpm for 5 minutes at 4 °C. Protein concentration in the supernatant was quantified by bicinchoninic acid assay (BCA) (Pierce, Rockford, IL). Ten µg of protein from RPE/choroid tissues was loaded into 4% to 4-12% NuPAGE Bis-Tris gels (Invitrogen, Carlsbad, CA) and transferred to a PVDF membrane (Invitrogen), and then incubated with antibody to Rap1a (1:1000, Abcam), VEGF (1:500, Santa Cruz Biotechnology, Santa Cruz, CA), caspase 3, LC3A/B, or phosphorylated NF-kB (Ser 536) (1:1000, Cell signaling Technology Inc., Danvers, MA) overnight at 4 °C. Membranes were reprobed with HRP-conjugated β-actin (Santa Cruz Biotechnology) as loading controls.

Densitometry was analyzed with the use of the software UN-SCAN-IT version 7.1 (Silk Scientific, Orem, UT).

### Statistics

Analysis of variance (ANOVA) was used to analyze protein expression and TUNEL positive cells to compare experimental and control groups, with one observation per animal or one well of cells from each treatment. Ordinary ANOVA requires that all data points, or observations, be independent, which is the case if only one observation is used per animal. When multiple observations are used per animal, and assumption is usually violated, since observations within the same animal tend to be more alike than they are between animals. The intraclass correlation coefficient (ICC) can be used to determine how correlated the observations are. If the ICC equals zero, then ordinary ANOVA provides a correct analysis. If ICC > 0, however, a method such as mixed effects linear regression is required^33,34^. This method is basically an ANOVA with an adjustment to the standard error to account for the lack of independence of the observations. For our CNV lesion outcome, we used mixed effects linear regression to account for lack of independence due to spots being clustered, or nested, with the same eye, with one eye per animal.

Results were displayed as Means ± SEM. A *P* value of ≤0.05 was considered statistically significant. For animal studies, at least 40 spots from 12 individual mice were analyzed for CNV volume. Retinal sections for GFP staining and western blots of Rap1 protein were taken from 3-6 different mice.

## Supplementary information

Supplementary information
