# Supplementary material for: Optimal Inhibition of Choroidal Neovascularization by scAAV2 with VMD2 Promoter-driven Active Rap1a in the RPE
Source: Sci Rep. 2019 Oct 31;9:15732. doi: 10.1038/s41598-019-52163-z (PMC6823539; doi:10.1038/s41598-019-52163-z)
Supplement: Supplementary file 1 — Supplementary information [file 41598_2019_52163_MOESM1_ESM.pdf]

Optimal Inhibition of Choroidal Neovascularization by scAAV2 with VMD2 Promoter-driven Active Rap1a in the RPE

Authors: Haibo Wang[1], Eric Kunz[1], Gregory J. Stoddard[2], William W. Hauswirth[3], M. Elizabeth Hartnett[1]*

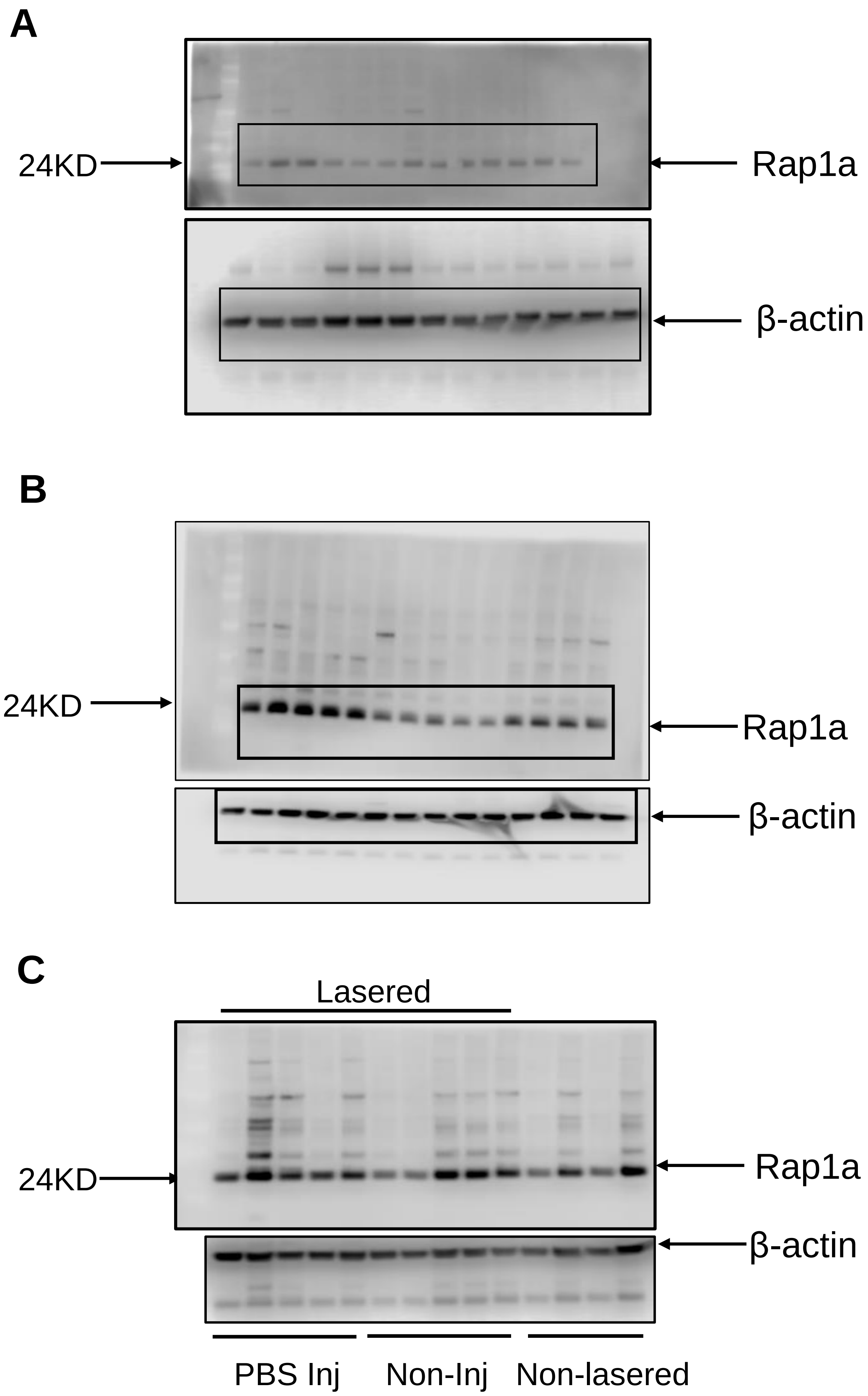

Supplemental Figure 1: (A-B) Full length gels for Rap1a and β-actin of Figure 3B and C (Boxes refer to the places from which the gels presented in Figure 3 were cropped); **(C)** Rap1a protein in RPE/choroids from 7 days post lasered mice injected with PBS (PBS Inj) or non-injected (Non-Inj), and non-lasered mice.

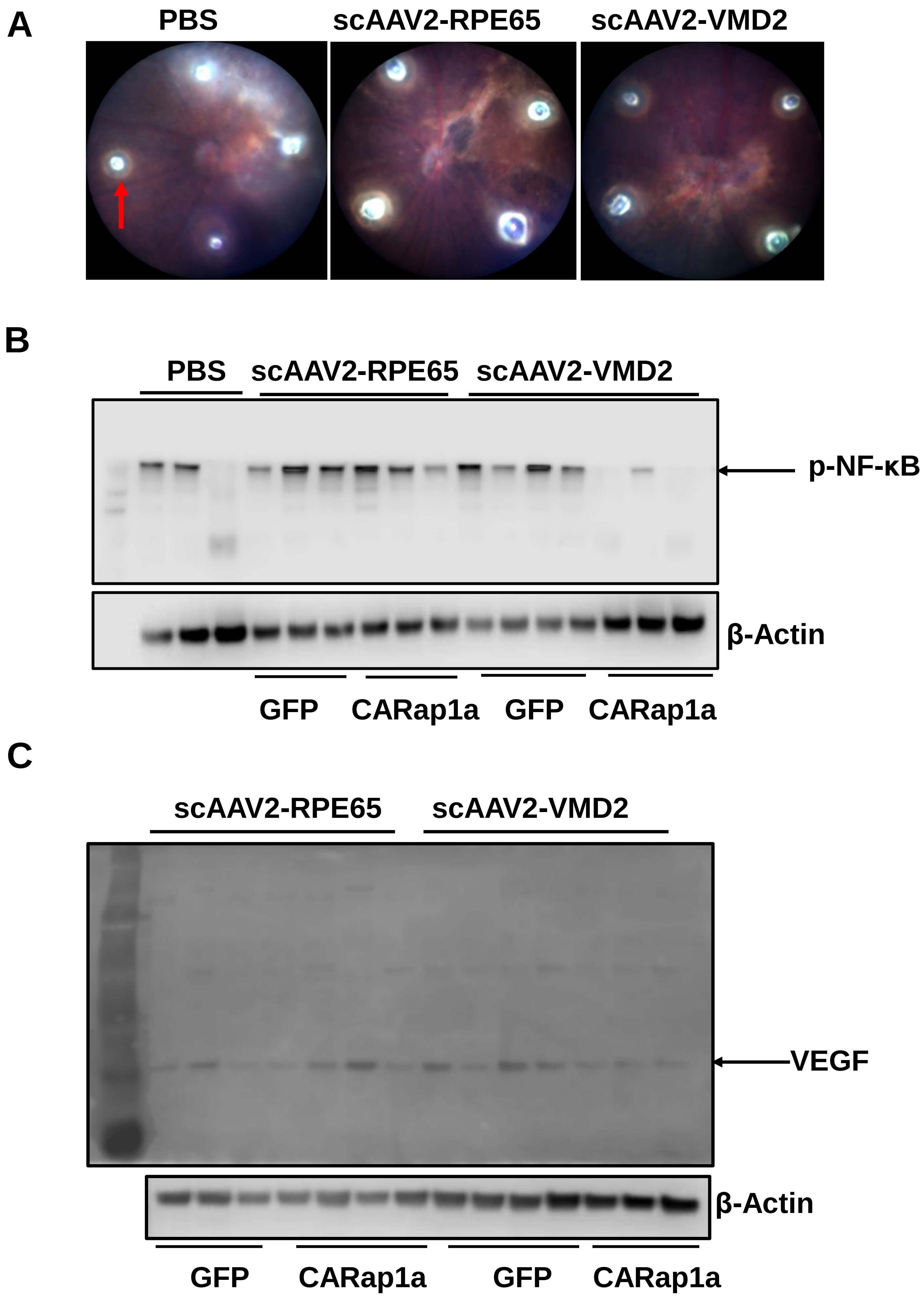

Supplemental Figure 2. **(A)** Fundus images taken during laser treatment (Arrow points to the spot with laser burn); Representative gel images of western blots of (**B)** p-NF-κB and actin and (**C**) VEGF in RPE/choroids from mice injected with PBS, scAAV2-RPE65 or scAAV2-VMD2 virus.

A

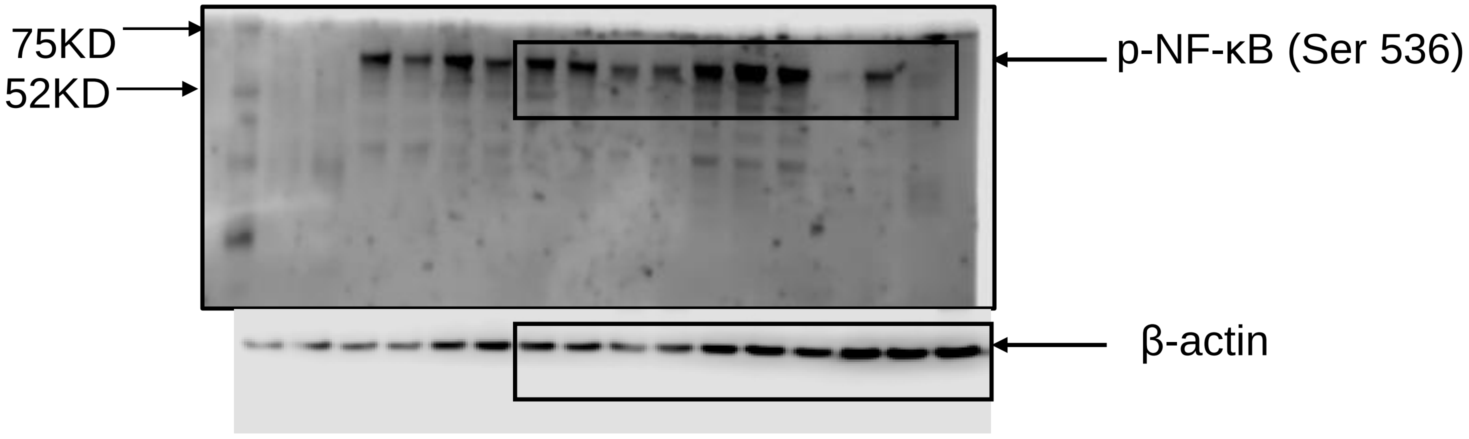

B

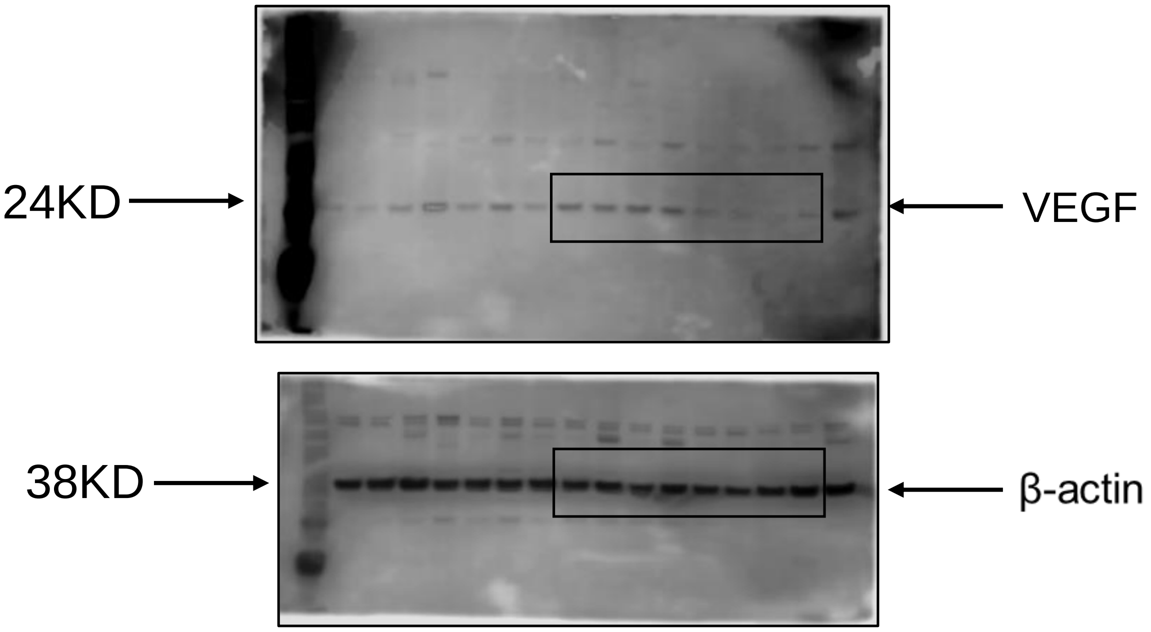

Supplemental Figure 3: Full length gels for (**A**) p-NF-κB and β-actin and (**B**) VEGF and β-actin (Boxes refer to the places from which the gels presented in Figure 5A and C were cropped).

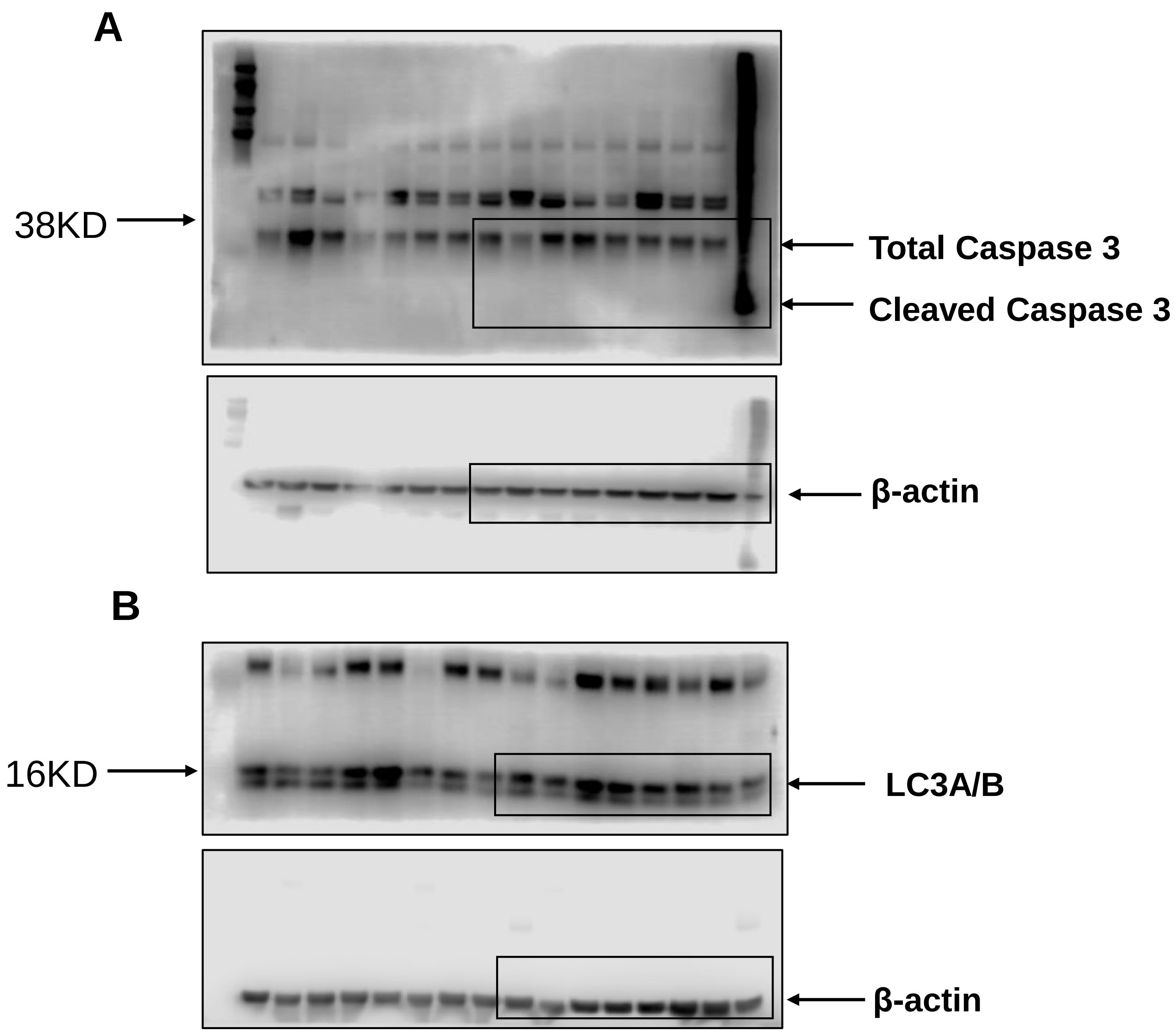

Supplemental Figure 4: Full length gels for (A) caspase 3 and β-actin and (B) LC3A/B and β-actin (Boxes refer to the places from which the gels presented in Figure 6A and C were cropped).

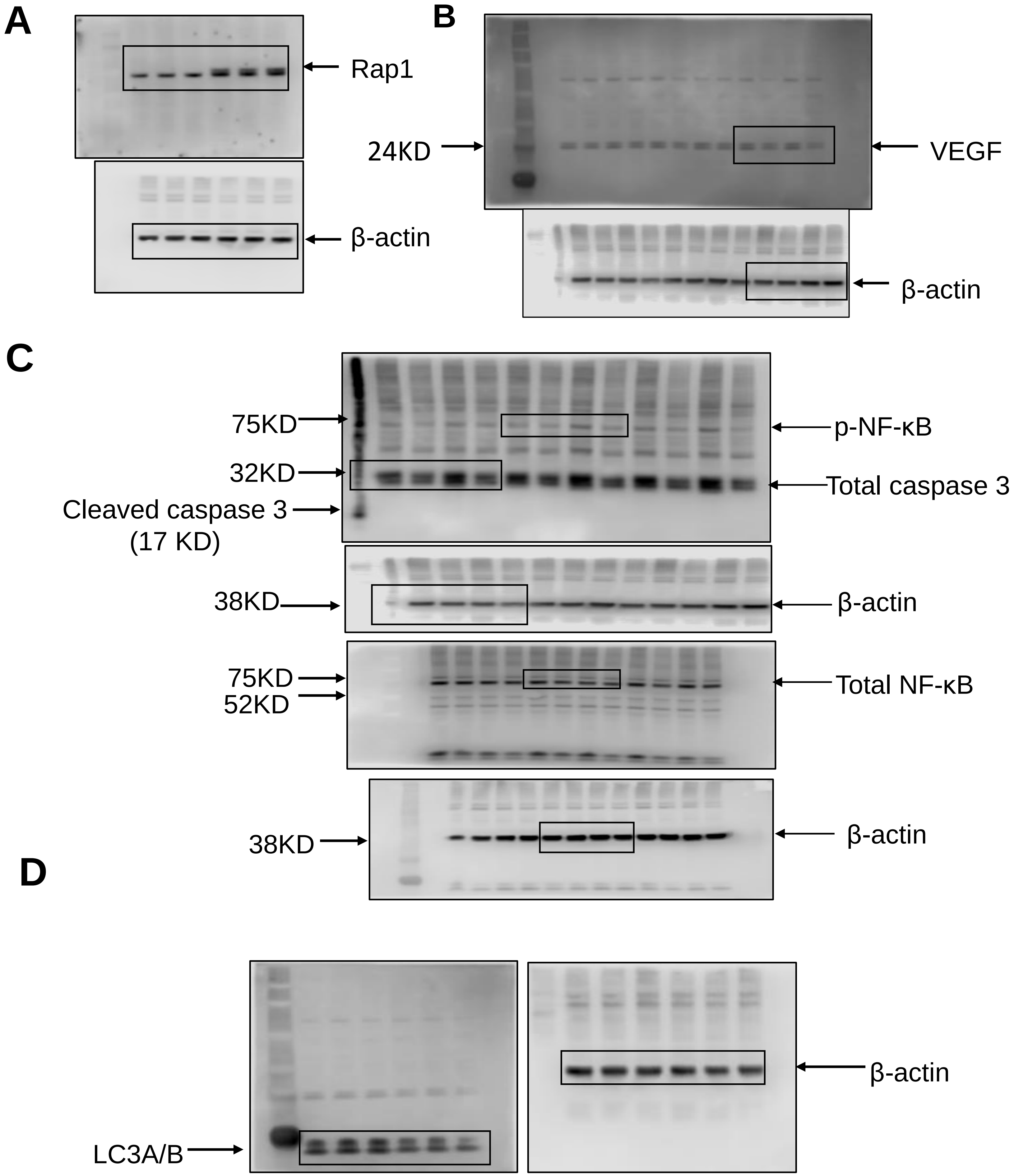

Supplemental Figure 5: Full length gels for (**A**) Rap1 and β-actin, (**B**) VEGF and β-actin, (**C**) p-NF-κB, total NF-κB, total caspase3 and β-actin and (**D**) LC3A/B and β-actin (Boxes refer to the places from which the gels presented in Figure 7B, D, E and Figure 8 A and B were cropped).
